# 

**Published:** 1882-06

**Authors:** 


### CONTENTS:

PAGE.

ORIGINAL COMMUNICATIONS:

Symptomatology of the Pupil. By Lucien Howe, M. D., . . . . . . 481
Rupture of the Perineum in Labor; Its Prevention and Treatment. By Matthew D. Mann, A. M., M. D., . . . . 498
An Objection to "Listerism." By C. M. Daniels, M. D., . . . . . . 512

CLINICAL REPORTS:

Compound Dislocation. By Dr C. C. F. Gay. Three Cases, . . . . 515

SELECTIONS:

Treatment of Epilepsy, . . . . . 517
The Therapeutics of Venesection, . . . 518
Risks of Intra-Pleural Injections, . . . 519

SOCIETY REPORTS:

Buffalo Medical and Surgical Association, . 520

EDITORIAL.

Medical Education, . . . . . . 524
Editorial Notes, . . . . . . . 526

REVIEWS, . . . . . . . 528

